# Supplementary figures and images for: Bacteriophages targeting protective commensals impair resistance against Salmonella Typhimurium infection in gnotobiotic mice
Source: PLoS Pathog. 2023 Aug 21;19(8):e1011600. doi: 10.1371/journal.ppat.1011600 (PMC10470868; doi:10.1371/journal.ppat.1011600)

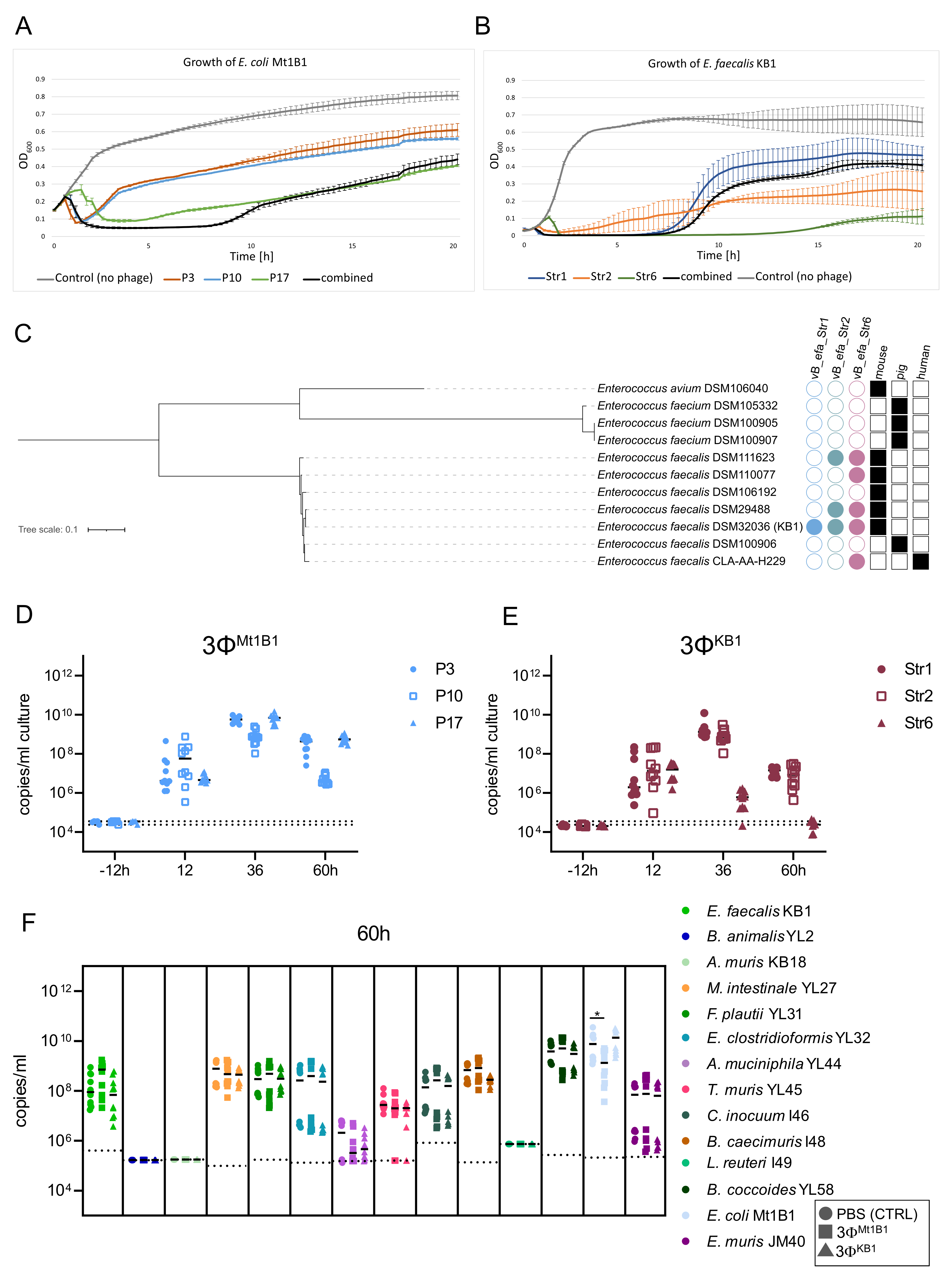

Supplement: S1 Fig — (A) Growth curve of E. coli Mt1B1 in LB medium (grey) measured under anaerobic conditions at 37°C, challenged with phage P3 (red), P10 (blue), P17 (green) and all three phages combined (black). (B) Growth curve of E. faecalis KB1 in BHI medium (grey) measured under anaerobic conditions at 37°C, challenged with phage Str1 (blue), Str2 (orange), Str6 (green) and all three phages combined (black). (C) Host range of phages Str1, Str2 and Str6 on different E. faecalis and E. faecium isolates, displayed on a phylogenetic tree of the bacteria. Filled circles stand for susceptibility, empty circles stand for resistance. (D) abundance of single phages of the phage cocktails 3ΦMt1B1 and (E) 3ΦKB1 in copies per ml batch culture from the experiment shown in Fig 1. Different shapes show the different phages, respectively. (F) Community composition 60h after phage addition from experiment shown in Fig 1, absolute abundance of each strain was determined using a strain-specific qPCR and plotted as 16S rRNA copy numbers per ml culture. Statistical analysis was performed using the Mann-Whitney Test comparing the treatment groups (N = 10) against the control group (N = 10) (* p<0.05, ** p<0.01, *** p<0.001). Each dot represents one well, black lines indicate median, dotted lines indicate detection limit (DTL). (TIFF) [file ppat.1011600.s001.tiff]

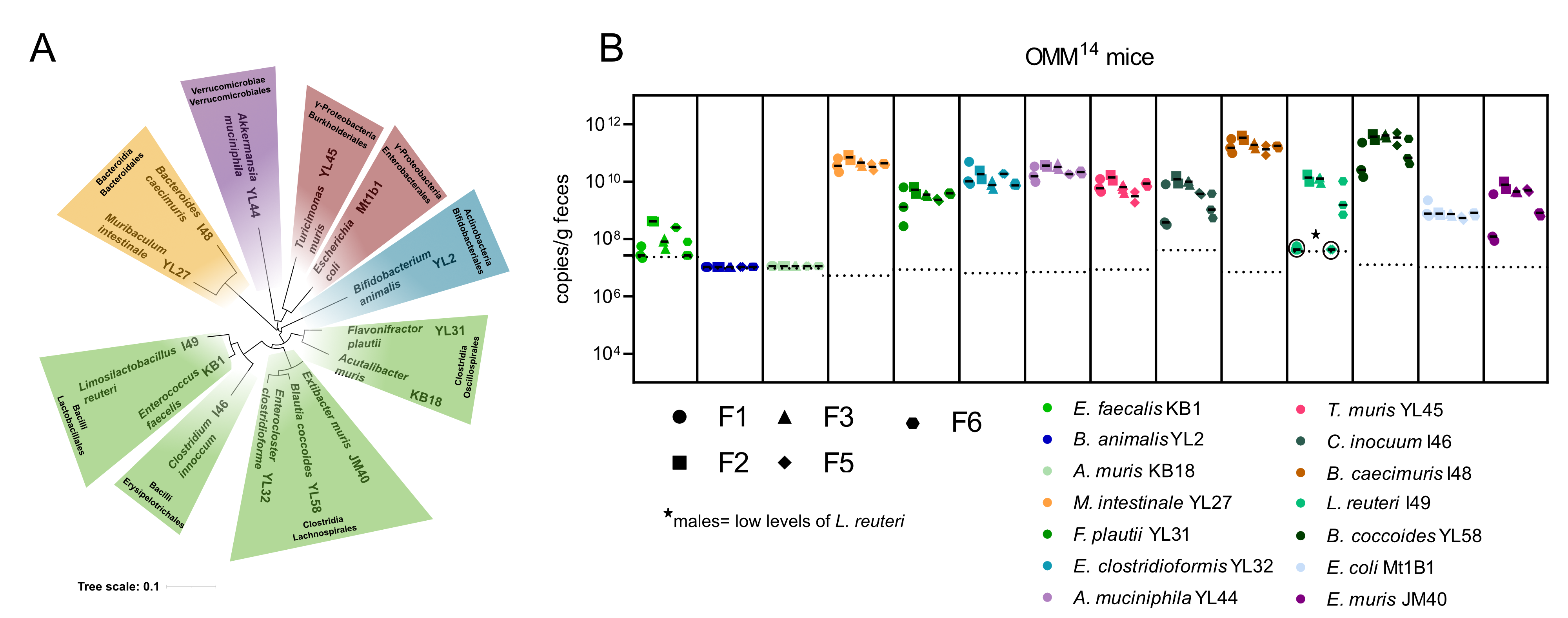

Supplement: S2 Fig — (A) Phylogenetic tree of the OMM14 bacterial community based on 16S rRNA sequences. Different colors represent different phyla. (B) Absolute abundance of all 14 bacteria overall several breeding generations (F1, F2, F3, F5 and F6, indicated by different shapes), determined by strain-specific qPCR in 16S rRNA copies per gram feces. Each dot represents one mouse, black line indicates median, dotted lines indicate DTL. (TIFF) [file ppat.1011600.s002.tiff]

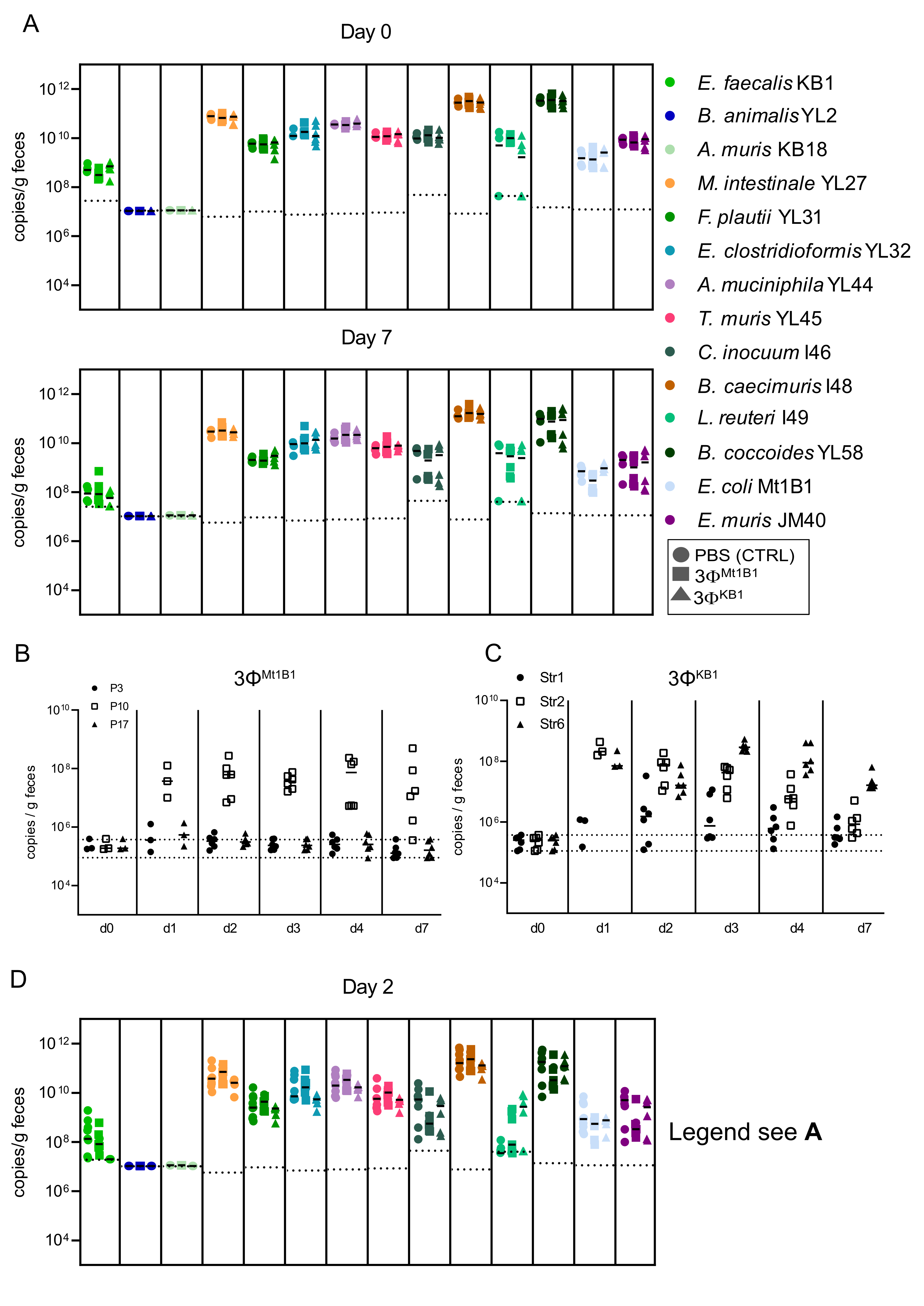

Supplement: S3 Fig — (A) Absolute abundances of all 14 bacteria from experiment shown in Fig 2, determined by strain-specific qPCR on day 0 and day 7 p. c.. Each color represents one bacterial strain, different shapes represent different experimental groups. (B) Abundances of the single phages of the phage cocktails 3ΦMt1B1 and (C) 3ΦKB1, determined by specific qPCR. Different shapes show different phages. (D) Absolute abundances of all 14 bacteria shown in Fig 3, determined by strain-specific qPCR on day 2 p. c.. Each color represents one bacterial strain, different shapes represent different experimental groups. Each dot represents one mouse, black line indicates median, dotted lines indicate DTL. (TIFF) [file ppat.1011600.s003.tiff]

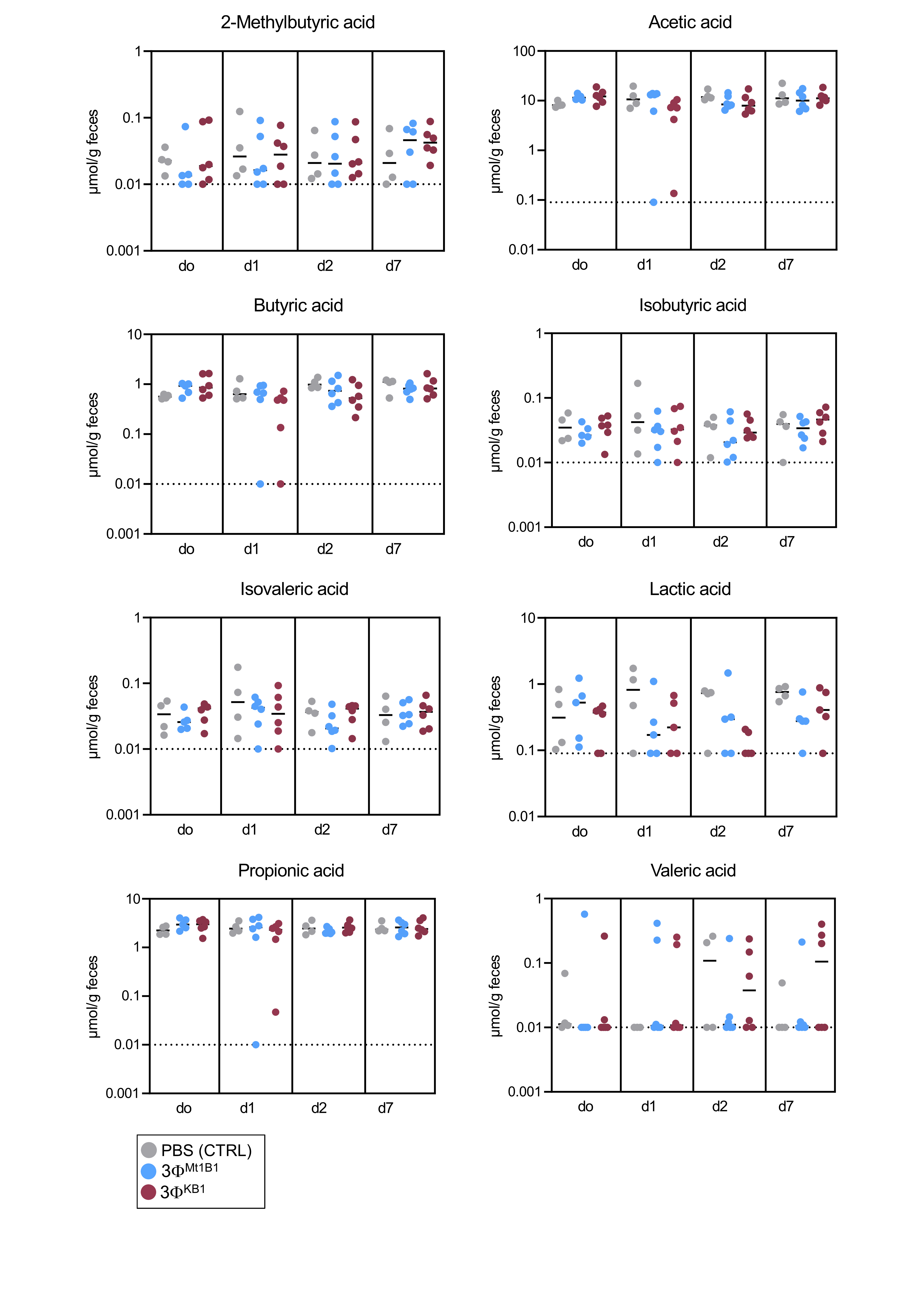

Supplement: S4 Fig — Different SCFAs were measured by quantitative mass spectronomy in fecal samples from the experiment shown in Fig 2. Different colors represent the different experimental groups, each dot represents one mouse, black line indicates median, dotted lines indicate DTL. (TIFF) [file ppat.1011600.s004.tiff]

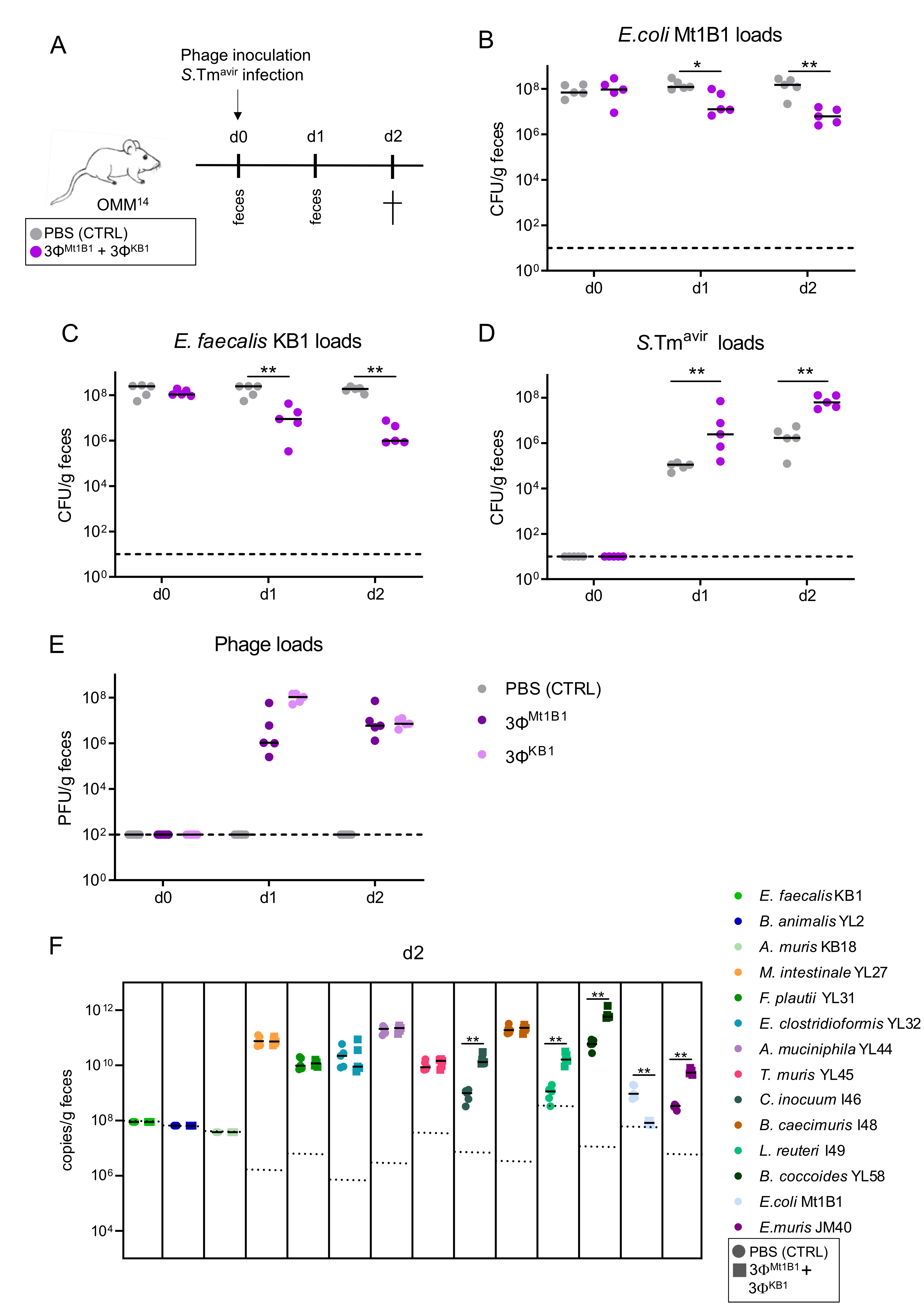

Supplement: S5 Fig — (A) Experimental setup: mice stably colonized with the OMM14 community were infected with S. Tmavir (5x10^7^ CFU) and directly after challenged orally with phage cocktails 3ΦMt1B1 and 3ΦKB1 (107 PFU per phage) or PBS as control. Feces were taken at day one and two p.c. and mice were sacrificed at day two p.c.. (B) E. coli Mt1B1 and (C) E. faecalis KB1 loads (CFU/g feces) were determined in feces by plating. (D) S. Tmavir loads at day 1 and 2 after phage challenge (= day 1 and 2 post infection (p.i.)). (E) Phage loads (PFU/g feces) were determined by spot assays. (F) Absolute abundances of all 14 bacteria, determined by strain-specific qPCR on day 2 p. c.. Each color represents one bacterial strain, different shapes represent different experimental groups. Statistical analysis was performed using the Mann-Whitney Test (* p<0.05, ** p<0.01, *** p<0.001, N = 5). Each dot represents one mouse, black lines indicate median, dotted lines indicate limit of detection. (TIFF) [file ppat.1011600.s005.tiff]

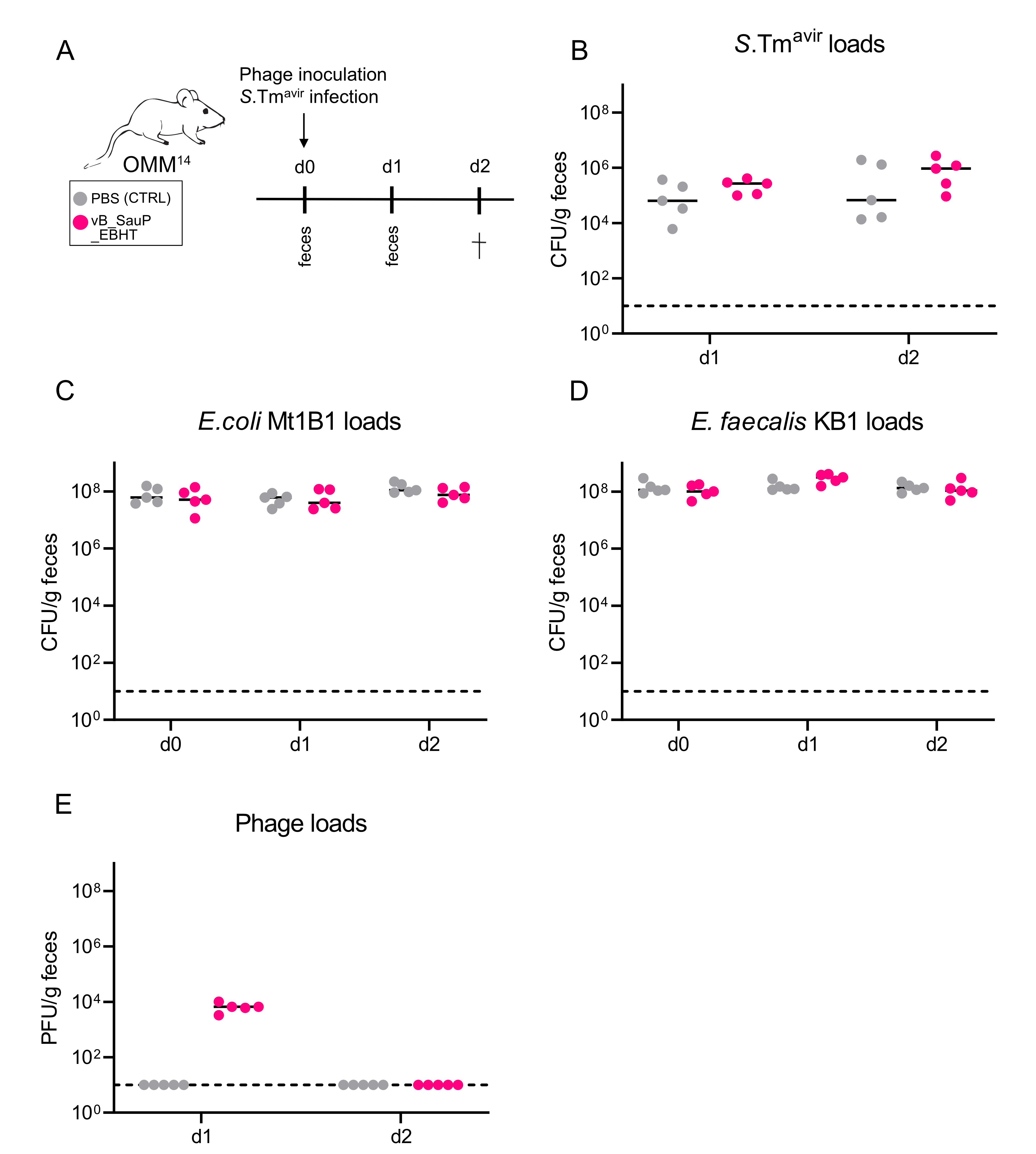

Supplement: S6 Fig — (A) Experimental setup: mice stably colonized with the OMM14 community were challenged orally with phage vB_SauP_EBHT (107 PFU) or PBS as a control and at the same time challenged with S. Tmavir (5x10^7^ CFU). Feces for plating were taken and mice were sacrificed on day 2 p. i.. (B) S. Tmavir loads, (C) E. coli Mt1B1 loads and (D) E. faecalis KB1 loads were monitored via plating. (E) phage loads were determined via spot assays on S. aureus. Each dot represents one mouse, black line indicates median, dotted lines indicate DTL. (TIFF) [file ppat.1011600.s006.tiff]

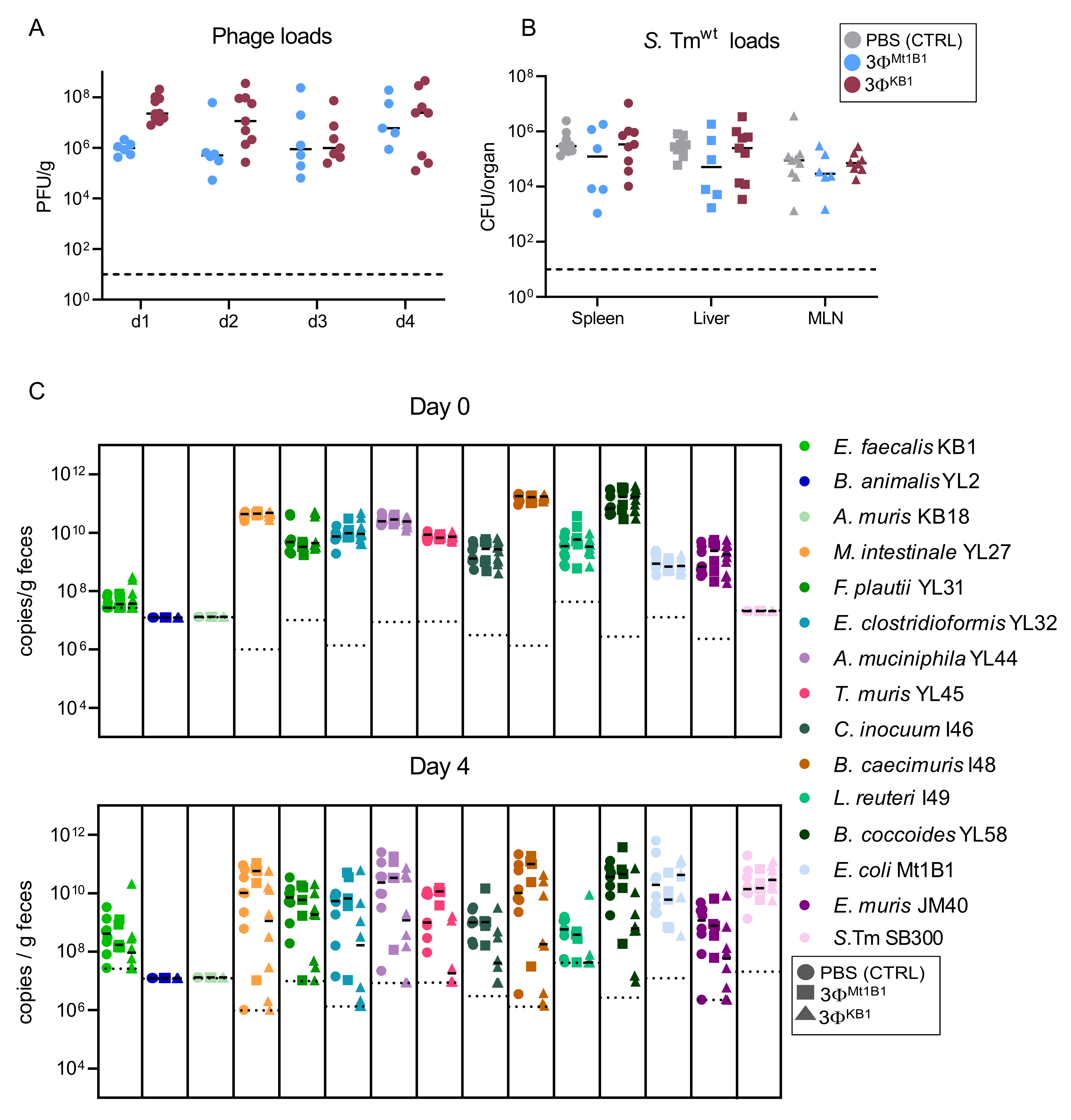

Supplement: S7 Fig — (A) Phage loads in feces from experiment shown in Fig 4, determined by spot assays. (B) S. Tmwt loads in spleen, liver and mesenteric lymph nodes (MLN), determined by plating. (C) absolute abundance of all 14 bacteria on day 0 and day 4 p. i. with S. Tmwt, determined by strain-specific qPCR. Each dot represents one mouse, black line indicates median, dotted lines indicate DTL. (TIFF) [file ppat.1011600.s007.tiff]
