# Supplementary material for: Bacteriophages targeting protective commensals impair resistance against Salmonella Typhimurium infection in gnotobiotic mice
Source: PLoS Pathog. 2023 Aug 21;19(8):e1011600. doi: 10.1371/journal.ppat.1011600 (PMC10470868; doi:10.1371/journal.ppat.1011600)
Supplement: Supplementary file 2 [file ppat.1011600.s008.docx]

Table S1

Designation	Sequence (5´- 3´)	Specificity	Reference
vB_efa_Str1_std_fwd	GGCAAGAACTTATGGAACA	vB_efa_Str1 (major capsid protein)	This study
vB_efa_Str1_std_rev	CTGGGTGTCAAAGTGATAA		
vB_efa_Str2_std_fwd	AACTTACTGGCAACTGAC	vB_efa_Str2 (major capsid protein)	This study
vB_efa_Str1_std_rev	TACCTTTTCTTCTGGCTCT		
vB_efa_Str6_std_fwd	TTTACCTCAATGTCCACC	vB_efa_Str6 (major tail protein)	This study
vB_efa_Str6_std_rev	TCTAGCTACATTCGTGGT		
Mt1B1_P3_fwd	GTCTGGCTTCGATTCTTT	Mt1B1_P3 (phage tail fibers protein)	This study
Mt1B1_P3_rev	GGCTTTTCTACTTCCCTG		
Mt1B1_P10_fwd	ATCCCACCTCCTTATGCT	Mt1B1_P10 (phage tail fibers protein)	This study
Mt1B1_P10_rev	GTACGCAAGTAACCTATCCC		
Mt1B1_P17_fwd	CTCGGTAACGTCCACACTA	Mt1B1_P17 (phage tail fibers protein)	This study
Mt1B1_P17_rev	TCGTTGTGGCTTACCTCT		
vB_efa_Str1_fwd_qPCR	AGAAACACGTGCATTACCAGAATC	vB_efa_Str1 (major capsid protein)	This study
vB_efa_Str1_rev_qPCR	TCTGGGATAATTGCTGATGCAT		
vB_efa_Str1_Probe	FAM-TTTGAAGGTGTTAAGTCTG-BHQ-1		
vB_efa_Str2_fwd_qPCR	GCCCTAAACAACTACAACCATGAA	vB_efa_Str2 (major capsid protein)	This study
vB_efa_Str2_rev_qPCR	CTGAAGACCAGTTCTCTCCCAAA		
vB_efa_Str2_Probe	HEX-TTGGTGCAGCTTGGA-BHQ1		
vB_efa_Str6_fwd_qPCR	CGCCTCGTTGTGCTGCTA	vB_efa_Str6 (major tail protein)	This study
vB_efa_Str6_rev_qPCR	CGTGGTACGGCAGTATTAATCG		
vB_efa_Str6_Probe	HEX-ATCCATTCGCCAAGGTCGTTCTGTACC-BHQ1		
P3_fwd_qPCR	GTAATCTGTGCGCCAGTCGTT	Mt1B1_P3 (phage tail fibers protein)	This study
P3_rev_qPCR	CAGGGCAGCGCACCAT		
P3_probe	FAM-AACTGGTGCCTTCACCTCCGCAAAA-BHQ1		
P10_fwd_qPCR	GCGATCGTGTACCAAGGGATA	Mt1B1_P10 (phage tail fibers protein)	This study
P10_rev_qPCR	GGATATTGAGATTGCTGGCCTTA		
P10_probe	HEX-TCTGTGCGCAATACCAGAAGTCATACCTGC_BHQ1		
P17_fwd_qPCR	GCGCAGACATGTGACTTGTAAAG	Mt1B1_P17 (phage tail fibers protein)	This study
P17_rev_qPCR	GATAACAACGAAGGAAGAACACCAA		
P17_probe	HEX-CGCAGCCACTTCTCCGTTGGGA_BHQ1		
