# Supplementary material for: Bacteriophages targeting protective commensals impair resistance against Salmonella Typhimurium infection in gnotobiotic mice
Source: PLoS Pathog. 2023 Aug 21;19(8):e1011600. doi: 10.1371/journal.ppat.1011600 (PMC10470868; doi:10.1371/journal.ppat.1011600)
Supplement: Supplementary file 3 [file ppat.1011600.s009.docx]

| Plasmid | Backbone | Origin of insert | Restriction site used for linearization | Reference |
|---|---|---|---|---|
| pAvS1 | pJET 1.2 | vB_efa_Str1 | NotI | This study |
| pAvS2 | pJET 1.2 | vB_efa_Str2 | NotI | This study |
| pAvS3 | pJET 1.2 | vB_efa_Str6 | NotI | This study |
| pAvS4 | pJET 1.2 | Mt1B1_P3 | NotI | This study |
| pAvS5 | pJET 1.2 | Mt1B1_P10 | NotI | This study |
| pAvS6 | pJET 1.2 | Mt1B1_P17 | NotI | This study |
